# Vasodilators for acute heart failure—A protocol for a systematic review of randomized clinical trials with meta‐analysis and Trial Sequential Analysis

**DOI:** 10.1111/aas.14130

**Published:** 2022-08-23

**Authors:** Johannes Grand, Olav W. Nielsen, Jacob Eifer Møller, Christian Hassager, Janus Christian Jakobsen

**Affiliations:** ^1^ Department of Cardiology Copenhagen University Hospital Hvidovre and Amager‐Hospital Copenhagen Denmark; ^2^ Department of Cardiology, Bispebjerg Hospital Copenhagen University Hospital Copenhagen Denmark; ^3^ Department of Clinical Medicine University of Copenhagen Copenhagen Denmark; ^4^ Department of Cardiology The Heart Center, Copenhagen University Hospital Rigshospitalet Copenhagen Denmark; ^5^ Department of Clinical Medicine University of Southern Denmark Odense Denmark; ^6^ Department of Cardiology University of Southern Denmark Odense Denmark; ^7^ Copenhagen Trial Unit, Centre for Clinical Intervention Research Copenhagen University Hospital – Rigshospitalet Copenhagen Denmark; ^8^ Department of Regional Health Research, Faculty of Health Sciences University of Southern Denmark Odense Denmark

**Keywords:** acute heart failure, emergency medicine, vasodilators

## Abstract

**Background:**

Above one million annual hospitalizations occur with a primary diagnosis of acute heart failure in the US, with comparable numbers in Europe. Within 1 year, over a third of patients have died or been re‐hospitalized. Most patients have acutely elevated systemic and/or intra‐cardiac blood pressures as part of the acute heart failure syndrome. Most clinical trials of acute heart failure have aimed at reducing preload and/or afterload through drug‐induced vasodilation. However, recent European guidelines downgraded the treatment recommendation of vasodilators. We aim to assess the beneficial and harmful effects of vasodilators in the treatment of acute heart failure.

**Methods:**

This protocol for a systematic review was undertaken using the recommendations of The Cochrane Collaboration and the Preferred Reporting Items for Systematic Reviews and Meta‐Analysis Protocols. We plan to include all randomized clinical trials assessing the use of vasodilators in the treatment of AHF. The systematic review will be conducted based on a systematic search of relevant major medical databases without date restrictions, including MEDLINE, Embase, and the Cochrane Central Register of Controlled Trials (CENTRAL) in addition to clinical trial registries. We will begin the searches in August 2022. All included trials will be assessed and classified at low risk of bias or at high risk of bias. Our conclusions will be based on the results from the primary outcomes with concomitant low risk of bias. Extracted data will be analyzed using Trial Sequential Analysis 0.9.5.10, Review Manager 5.3, and SAS. We will assess the certainty of the evidence using the Grading of Recommendations Assessment, Development and Evaluation. We will register this systematic review at Prospero and aim to update it when new trials are published.

**Discussion:**

This protocol defines the detailed methodology and approach used for a systematic review on whether vasodilation for acute heart failure improves patient outcome. This systematic review will potentially aid clinicians in deciding the optimal treatment of patients admitted with acute heart failure. Furthermore, this review will explore gaps in our knowledge and thus guide future research within acute heart failure.

## 1 INTRODUCTION

### 1.1 Acute heart failure

#### 1.1.1 Epidemiology

Up to a third of patients hospitalized with acute heart failure will die or be re‐hospitalized within 3 months.^1^, ^2^, ^3^, ^4^ Acute heart failure is a leading cause of hospitalizations in patients aged >65 years and over one million emergency department hospitalizations occur each year with a primary diagnosis of acute heart failure in the USA, with comparable numbers in Europe.^5^, ^6^ In the United States, heart failure is the most expensive reason for admission and re‐admission for older patients.^7^ With increasing age and increasing prevalence of obesity, this burden will likely continue to rise.^8^ The poor outcomes for acute heart failure stand in contrast to the progress made in other cardiovascular fields, such as chronic heart failure and acute coronary syndrome.^9^

#### 1.1.2 Definition and classification

Heart failure is a chronic syndrome characterized by symptoms and clinical findings such as dyspnea, pulmonary, and systemic congestion.^10^ The syndrome is a consequence of myocardial dysfunction or structural cardiac disease that may lead to either of or a mixture of forward and backward failures.^10^ An acute heart failure episode is seen when heart failure presents itself de novo with abrupt symptoms leading to an emergency department visit or when patients with known heart failure have an acute exacerbation or worsening of symptoms requiring hospitalizing. Acute heart failure refers to rapid onset of symptoms and clinical signs of heart failure leading to unplanned hospital admission or an emergency department visit.^10^ Patients with acute heart failure should be evaluated immediately and subsequent treatment administered in the acute setting within the first few minutes to hours.^10^

Four main clinical presentations are emphasized in the 2021 European guidelines for heart failure. The four phenotypes can be separated based on the presence of signs of congestion (no congestion = dry; congestion = wet) and/or peripheral hypoperfusion (normoperfusion = warm; hypoperfusion = cold) and may require different treatments.^10^ Acutely decompensated heart failure is the most common form of acute heart failure, accounting for 50%–70% of presentations.^11^ Acute pulmonary edema occurs in 13%–25% of presentations.^12^ Less than 5% present with isolated right ventricular failure or cardiogenic shock.^11^, ^12^ The division into phenotypes is mostly academic and guidelines emphasize that overlaps between them should be considered.

#### 1.1.3 Pathophysiology

More than 75% of acute heart failure presents as warm and wet with predominantly acute backward failure of the ventricles and adequate perfusion.^11^ Even though both hypertensive pulmonary edema and acute decompensated heart failure are both “warm and wet,” they differ significantly in clinical presentation.

The pathophysiology of acute hypertensive pulmonary edema (warm and wet) is presumed started by an external trigger leading to increased systemic vascular resistance and increased systemic blood pressure.^13^ Sustained increased blood pressure elevation and arterial stiffness increase LV afterload.^14^ In the young and healthy heart, the left ventricle compensates for an increased afterload by using preload reserve and increase end‐diastolic volume.^13^ In patients with diastolic dysfunction or reduced ejection fraction, even small increases in LV end‐diastolic volume may be associated with a significant elevation in LV end‐diastolic pressure.^13^ Pressure backs up from the left ventricle to the left atrium resulting in increased pulmonic blood pressure and retention of fluids in the lungs.^15^ Anxiety and hypoxemia further worsen the condition in the clinical setting. Most patients presenting with pulmonary edema have preserved systolic LV function. However, often the evaluation of LV systolic function occurs after the pulmonary edema has been treated. One study of patients in the acute phase of hypertensive pulmonary edema found that the pulmonary edema was due to exacerbation of diastolic dysfunction and not a transient systolic dysfunction.^16^

Acute decompensated heart failure (warm and wet) is dominated by systemic fluid retention in relation to fluid and salt retention. An initial theory was that left ventricular backward failure increased pulmonic pressures and consequently right ventricular afterload leading the progressive right ventricular backward failure. However, more likely forward failure of the left ventricle activates the renin‐angiotensin‐aldosterone system with stimulation of renal tubular reabsorption of sodium and water.^17^ Isolated right ventricular failure is rare and causes blood to back up in the central venous system with congestion of liver, kidneys, and intestines, peripheral edema, and elevated jugular venous pressure.^18^

Cardiogenic shock (cold and wet/dry), involving 2%–5% of acute decompensated heart failure cases, is associated with severe forward failure of the left ventricle with consequently decreased cardiac output by means of either impaired myocardial function, acute mechanical failure of the cardiac structural integrity, or both.^19^ Symptoms of severe forward failure are life threatening and related to organ hypoperfusion with confusion, decreased urine output, and increased lactate.^20^, ^21^

This systematic review will focus on the “warm and wet” syndromes of acute heart failure, whereas cardiogenic shock and isolated right ventricular failure will be out of the scope.

### 1.2 Description of the intervention

European Society of Cardiology (ESC) 2021 guidelines for acute heart failure recommend three therapies to be commenced: first, oxygen and continuous positive airway pressure/non‐invasive positive‐pressure‐ventilation. Second, intravenous loop‐diuretics. Third, intravenous vasodilators *may be considered* (IIb) when SBP is >110 mmHg in case of acute heart failure with pulmonary edema or congestion.^10^ In case of hypertension, blood pressure should be reduced by up to 25% using vasodilators and loop‐diuretic.^22^, ^23^ The 2017 ACC/AHA guidelines recommend reducing the systolic blood pressure to a maximum of 25% within the first hour; then, if the patient is clinically stable, lower the blood pressure to 160/100 mmHg over the next 2–6 h, and then cautiously to normal values over the following 24–48 h.^24^ The evidence supporting these strategies are sparse, since the effects of blood pressure‐lowering drugs, including loop‐diuretics and vasodilators, have been studied in few controlled clinical trials.^25^ Traditional treatments throughout the last 40 years have until recently been a combination of loop‐diuretics (often furosemide) and vasodilators (often nitrates).^26^

#### 1.2.1 Medical treatment in the emergency setting of acute heart failure without shock

Intravenous furosemide promotes the elimination of salt and water, resulting in a decreased overall intravascular volume.^27^ Diuresis occurs after 30–120 min, thus less helpful in the hyperacute setting of respiratory failure.^28^, ^29^, ^30^, ^31^, ^32^ However, a faster effect on venodilation has been reported after 15 min, thus decreasing preload of both ventricles.^28^ But furosemide also activates the sympathetic and the renin‐angiotensin‐aldosterone systems, increasing systemic vascular resistance,^33^ which may increase left ventricular‐afterload and decrease cardiac output. Furthermore, Kraus et al.^34^ demonstrated that left ventricular preload (pulmonary capillary wedge pressure [PCWP]) paradoxically increases in the first 20 min after administration of furosemide. This increase in PCWP was attributed to a documented increase in renin activity as well as increased noradrenaline and vasopressin levels prior to diuresis, which was found by Francis et al.^33^ in 15 patients with chronic heart failure, who saw an increase in systemic blood pressure after administration of intravenous furosemide. The resultant early vasoconstriction was their explanation for the PCWP increase in the first 20 min after administration of furosemide. From a physiological point of view, reduction of preload and afterload of the left ventricle is central for breaking the vicious circle of pulmonary congestion in the acute phase.^22^ Because of their mechanisms of action, it can be hypothesized that intravenous vasodilators may be more effective than intravenous diuretics in the emergency setting of acute heart failure with pulmonary congestion.

#### 1.2.2 Vasodilators

The hemodynamic effects of vasodilators vary considerable among drugs. Some vasodilators such as nitrates primarily work on the venous side of the circulatory system by a redistribution of the circulating blood volume away from the heart to the venous capacitance system.^35^ Consequently, the venous return to the heart decreases as well as preload for both ventricles. Intravenous nitrates cause potent nitric oxide‐induced vasodilation and exert effect within 2–3 min after administration.^28^, ^29^, ^30^, ^31^ The immediate effects include venous dilatation, redistribution of blood volume, reduction in venous return, less congestion, and a consequent relief of symptoms.^36^ Higher doses of nitrates in addition to sodium nitroprusside dilate arterial vessels, thus directly decreasing blood pressure and left ventricular afterload. The afterload reduction from the arterial effects of nitrates also decreases myocardial oxygen consumption and lowers intra‐ventricular systolic and diastolic pressures.^35^

Because of their mechanisms of action, intravenous vasodilators are thought to be effective in patients with acute heart failure and acute pulmonary edema, where increased cardiac and systemic blood pressures are severely elevated in the absence or with minimal systemic fluid accumulation.^3^, ^10^ Isosorbiddinitrat is an Intravenous nitrate with a half‐life of 29 min. The active metabolites have, furthermore, a half‐life up to 1.7 and 7.5 h ensuring a potentially stabilizing effect over many hours.^37^ Cotter et al.^25^ compared vasodilation (isosorbide dinitrate) with loop‐diuretics for treatment of acute heart failure with pulmonary edema and found vasodilation to be superior. However, a recent randomized trial, The GALACTIC trial, comparing usual care (including possible use of nitrates) with early intensive and sustained vasodilation and found no beneficial effect of vasodilators.^38^ Based primarily on this study, the cluster‐randomized ELISABETH study, nitrates were downgraded from IA to IIb in the 2021 guidelines for heart failure.^10^, ^39^ However, the GALACTIC trial did not include patients in the emergency setting in the emergency department with median time from emergency department presentation to randomization above 5 h.

So far, no vasodilator is given a guideline recommendation for acute heart failure other than IIb and “may be considered.” One possible shortfall of vasodilator trials is the time from emergency department presentation to randomization (sometimes >11 h [median] from admission).^38^, ^40^, ^41^, ^42^

### 1.3 Why is it important to do this review?

Numerous studies of several vasodilators have been published through the years and several reviews of both observational and randomized trials have assessed the effect of vasodilators.^43^, ^44^, ^45^, ^46^, ^47^ Some reviews have focused on single vasodilators^43^, ^47^ or have focused only on studies where pulmonary artery catheters are used.^44^ However, none of the reviews adhered to the Preferred Reporting Items for Systematic reviews and Meta‐analyses (PRISMA) guidelines, none of them published a protocol, none of them used Trial Sequential Analysis to minimize the risk of random errors, and only the 2013 review by Wakai et al.^46^ assessed the risk of bias in individual trials according to The Cochrane Collaboration risk of bias tool. There is a need for a systematic review assessing the harms and benefits of vasodilators for acute heart failure, adhering to the PRISMA statement, searching relevant databases, minimizing the risk of random error by a Trial Sequential Analysis, assessing the risk of bias in each trial, and assessing the certainty of the evidence with Grading of Recommendations Assessment, Development and Evaluation (GRADE).^48^, ^49^, ^50^

### 1.4 Review questions

The purpose of this systematic review is to summarize existing evidence of vasodilation for acute heart failure.

The questions sought to be answered are as follows.

1. In patients with acute heart failure, does vasodilators compared with placebo or no treatment benefit any clinical outcomes or induce any harms?
2. In patients with acute heart failure, is any vasodilator compared to other vasodilators superior in terms of effect size and/or harms.
3. Does treatment effect vary according to the specified subgroups?
4. Is there an interaction between time from presentation to treatment and effect of the treatment?

## 2 METHODS

This protocol for a systematic review is developed with guidance from the Cochrane Handbook^48^ and the Preferred Reporting Items for Systematic Reviews and Meta‐Analysis Protocols (PRISMA‐P).^49^

### 2.1 Criteria for including studies in this review

#### 2.1.1 Types of studies

We will include randomized clinical trials irrespective of setting, publication year, publication type, and language. We will also include cluster‐randomized trials. We will not include quasi‐randomized studies or observational studies.

#### 2.1.2 Types of participants, interventions, and outcome

1. Population: adults (≥18 years [or as defined in individual studies]) with acute heart failure (as defined by trialists) irrespective of age, sex, and comorbidities. Acute heart failure may be defined as new onset or worsening of symptoms and signs of heart failure leading to hospitalization in the presence of an underlying structural or functional cardiac dysfunction.^51^ We will also include other definitions of acute heart failure, if included studies use a different definition of acute heart failure. The trial population should consist of at least 80% patients with AHF to be included.
2. Interventions: drugs with a vasodilator effect, where the vasodilatory effect is used for potential benefit in the relevant patient group. A vasodilator may be defined as a medical drug with the ability to act as blood vessel dilators by relaxing their muscular walls such as intravenous nitrates (nitroglycerin, isosorbide mononitrate, isosorbide dinitrate, sodium nitroprusside, serelaxin, nesiritide, enalaprilat, hydralazine, clevidipine, inhibitors of the RAAS, calcium blockers). We recognize that individual studies might have used different definitions of vasodilators and whether individual studies are eligible for inclusion will be determined on a case‐by‐case basis. Inodilators such as levosimendan, dobutamine, and milrinone will not be included.
3. Comparators: trials will be included if the intervention is compared against placebo, no‐intervention, or alternatively against another active treatment. We plan to report the results of vasodilators versus placebo/no intervention and vasodilators versus active comparators separately.
4. Outcomes: the primary outcome is all‐cause mortality. Secondary outcomes will be the proportion of participants with one or more serious adverse events. We will use the International Conference on Harmonization of technical requirements for registration of pharmaceuticals for human use—Good Clinical Practice (ICH‐GCP) definition of a serious adverse event, which is any untoward medical occurrence that resulted in death, was life‐threatening, required hospitalization or prolonging of existing hospitalization, and resulted in persistent or significant disability or jeopardized the participant. If the trialists do not use the ICH‐GCP definition, we will include the data if the trialists use the term “serious adverse event.” If the trialists do not use the ICH‐GCP definition nor use the term serious adverse event, then we will also include the data, if the event clearly fulfills the ICH‐GCP definition for a serious adverse event. We will exploratorily assess each type of serious adverse event separately.

Other secondary outcomes will be health‐related quality of life (any valid continuous scale), and need for tracheal intubation.

Exploratory outcomes will be (1) days alive and out‐of‐hospital to Day 30; (2) NT‐Pro‐BNP; (3) blood pressure after intervention (continuous outcome); (4) ejection fraction (continuous outcome); (5) dyspnea (continuous outcome); (6) time to stabilization; (7) renal replacement therapy (dichotomous outcome); and (8) intubations.

For outcomes, we will use the study results reported at the longest follow‐up.

## 3 SEARCH METHODS

### 3.1 Information sources

We will search the following electronic bibliographic databases: Medline, Embase, Cochrane Central Register of Controlled Trials. The bibliographies of included articles will be reviewed for potential additional articles. We will begin the search in August 2022.

### 3.2 Searching other resources

The reference lists of relevant publications will be checked for randomized trials. We will contact the authors of included studies by email asking for unpublished randomized trials. Further, we will search for ongoing trials on the following.

1. ClinicalTrials.gov (www.clinicaltrials.gov).
2. Google Scholar (https://scholar.google.dk/).
3. The Turning Research into Practice (TRIP) Database (https://www.tripdatabase.com).
4. European Medicines Agency (EMA) (https://www.ema.europa.eu/ema/).
5. US Food and Drug Administration (FDA) (www.fda.gov).
6. China Food and Drug Administration (CFDA) (http://eng.sfda.gov.cn/WS03/CL0755/).
7. Medicines and Healthcare products Regulatory Agency (https://www.gov.uk/government/organisations/medicines-and-healthcare-products-regulatory‐agency).
8. The World Health Organization (WHO) International Clinical Trials Registry Platform (ICTRP) search portal (http://apps.who.int/trialsearch).
9. Clinical Trials Registry Platform (http://www.who.int/ictrp/en/).

### 3.3 Selection process

At least two reviewers, using pre‐defined screening criteria, will independently screen all titles and abstracts retrieved from the systematic searches in duplicate using dedicated software: Covidence (Covidence systematic review software, Veritas Health Innovation, Melbourne, Australia; available at www.covidence.org). Any disagreement regarding inclusion or exclusion will be resolved via discussion between the reviewers and with a third reviewer if needed. At least two reviewers will then review, in duplicate, the full‐text reports of all potentially relevant publications passing the first level of screening. Any disagreement regarding eligibility will be resolved via discussion and study authors will be contacted if pertinent. The final report will include a PRISMA diagram showing the number of studies remaining after each stage of the selection process. This will include reasons for the exclusion of full‐text articles.

### 3.4 Data extraction and management

RevMan (The Cochrane Collaboration, 2014) will be used to perform meta‐analysis of the study data and Trial Sequential Analysis.^50^ SAS software version 9.4 (SAS Institute) will be used if Revman is insufficient for the needed analysis. Two authors will in duplicate extract data from included trials. Disagreements will be resolved via discussion with a third author. Duplicate publications and publications from the same main trial will be assessed to evaluate all available data simultaneously and maximize data extraction. We will contact authors by email to expand any additional data, which might not have been reported sufficiently or at all in the publication.

## 4 TRIAL CHARACTERISTICS

### 4.1 Data items

The following data will be extracted as relevant:

1. General information
  - First author name
  - Year of publication
  - Geographical location of the study (country, continent)
  - Study design
  - Inclusion and exclusion criteria
  - Years of patient enrollment
  - Number of patients screened and analyzed (sample size)
  - Intervention/exposure/comparator
  - Time from admission to intervention
  - Length of follow‐up
2. Participants
  - Summary demographics
    - Age (mean/median)
    - Sex (proportion of females)
  - Type of acute heart failure (decompensated heart failure, pulmonary edema, cardiogenic shock)
  - Type of HF: HfrEF, HFpEF
  - Etiology of the acute heart failure arrest (de novo or worsening of chronic heart failure)
  - Baseline blood pressure
  - Baseline left ventricular ejection fraction
  - Baseline comorbidities, including atrial fibrillation or flutter, hypertension, baseline number of participants with heart failure, estimated glomerular filtration rate, and NT‐proBNP
  - Baseline heart failure medication (beta‐blockers, intravenous nitrates, intravenous loop‐diuretics, angiotensin‐converting enzyme inhibitors, angiotensin II‐receptor antagonists, and/or mineralocorticoid receptor antagonists)
3. Relevant results

### 4.2 Vasodilator strategy characteristics

Dose of intervention, timing of intervention, mode of administration, and duration of administration.

### 4.3 Co‐intervention characteristics

Type of co‐intervention, timing of co‐intervention, dose of co‐intervention, duration of co‐intervention, and mode of administration.

### 4.4 Risk of bias in individual studies

Our bias risk assessment will be based on the Cochrane Risk of Bias tool—version 2 (RoB 2) as recommended in The Cochrane Handbook of Systematic Reviews of Interventions.^52^

At least two investigators will independently assess the risk of bias for the included studies. The risk of bias will be assessed by use of the revised Cochrane risk‐of‐bias tool for randomized trials. For controlled trials, the assessment tool for individually randomized parallel‐group trials and the supplement for cluster‐randomized parallel‐group trial will be used as appropriate. We will evaluate the methodology in respect of the following.

1. Random sequence generation
2. Allocation concealment
3. Blinding of participants and treatment providers
4. Blinding of outcome assessment
5. Incomplete outcome data
6. Selective outcome reporting
7. For profit bias
8. Other risks of bias
9. Overall risk of bias

These components enable the classification of trials into being at overall “low risk of bias,” only if all bias domains is classified as “low risk of bias.” A trial will be classified as overall “high risk of bias,” if at least one of the bias domains are classified as “unclear” or “high risk of bias.” We will also evaluate for “profit bias” as specified in Data S1.

We will assess outcomes into the following categories: “blinding of outcome assessment,” “incomplete outcome data,” and “selective outcome reporting” so the bias risk for each outcome can be assessed. Our main conclusions will be founded on results of outcomes at overall low risk of bias.

In case of overlap in data between studies included in the meta‐analyses, the risk of bias within the individual studies will be compared and the study with the least risk of bias will be included. If the risk of bias is comparable, we will include the study with the largest sample size.

## 5 MEASURES OF TREATMENT EFFECT

For dichotomous outcomes, risk ratios (RRs) with 95% confidence interval (CI) will be calculated. For continuous outcomes, the mean differences (MDs) with 95% CI for continuous outcomes will be calculated. In the primary analysis, we will use variables assessed at single time points. If only changes from baseline are reported, we will analyze the results together with follow‐up scores. If standard deviations (SDs) are not reported, they will be calculated using trial data, if possible.

### 5.1 Missing data

We will use intention‐to‐treat data if such data are available. We will, if possible, contact trial authors to acquire relevant missing data (i.e., for data extraction and for assessment of risk of bias). We will not impute missing data for outcomes in our primary analysis, but imputations will be used in sensitivity analysis.

### 5.2 Sensitivity analysis

The potential impact of missing data will be assessed by the two following sensitivity analyses on both the primary and secondary outcomes.

“Best‐worst‐case” scenario: we will assume that all participants lost to follow‐up in the vasodilator groups have survived without any adverse events. We will assume the opposite for all participants lost to follow‐up in the control group.

“Worst‐base‐case” scenario: we will assume that all participants lost to follow‐up in the vasodilator group have not survived, with all adverse events. We will assume the opposite for all participants lost to follow‐up in the control group.

### 5.3 Heterogeneity

Studies will be assessed for clinical (i.e., participants, interventions, and outcomes), methodological (i.e. study design or risk of bias), and potentially statistical heterogeneity.^9^ If there is no substantial clinical or methodological heterogeneity, statistical heterogeneity will primarily be assessed via visual inspection of forest plots. A *p* value of <.10 or *I* ^2^ statistic of >50% will indicate considerable statistical heterogeneity.^9^ Possible heterogeneity will be investigated via sensitivity analyses and subgroup analyses. Ultimately, it may be decided that a meta‐analysis should be avoided because of unexpected high heterogeneity (clinical, methodological, or statistical). A narrative synthesis will be performed if heterogeneity is deemed too substantial between studies to allow for meaningful meta‐analyses.

### 5.4 Assessment of reporting biases

We will use a funnel plot for a visual evaluation of reporting bias, but only if 10 or more trials are included. We are aware of the limitations of a funnel plot (a funnel plot evaluates bias from trials with small sample sizes). From this information, we quantify possible reporting bias. We will test asymmetry with the Harbord test^53^ if τ2 is less than 0.1 and with the Rücker test if τ2 is greater than 0.1, for dichotomous outcomes. We will use the regression asymmetry test and the adjusted rank correlation test for continuous outcomes.^54^, ^55^

### 5.5 Units

For trials using a crossover design, only data from the first period will be included.^56^ We will also include cluster‐randomized trials after adjusting the original sample size to the effective sample size using the intra‐cluster correlation coefficient from the “design effect.”^48^ We, therefore, do not expect any unit of analysis issues.

## 6 DATA SYNTHESIS

### 6.1 Meta‐analysis

We will carry out meta‐analyses according to the international recommendations^48^ and the eight‐step assessment by Jakobsen et al.^57^ We will evaluate our intervention effects with both fixed effects meta‐analyses^58^ and fixed effects meta‐analyses.^59^ We will primarily report the most conservative results (highest *p* value) and the less conservative result will be considered a sensitivity analysis. We use one primary outcome and our primary conclusions will be based on this outcome, and therefore, we will consider a *p* value of .05 as the threshold for statistical significance for all outcomes. Our main conclusion will be based on the results from the primary outcomes at low risk of bias.

### 6.2 Trial Sequential Analysis

A detailed description of Trial Sequential Analysis is found in the Trial Sequential Analysis manual.^50^ Traditional meta‐analyses have a risk of random errors owing to sparse data and repeat testing of accumulative data when updating reviews. We seek to control the risks of Type I and II errors and therefore we will perform a Trial Sequential Analysis on the outcomes, to estimate the required information size (the number of participants needed in a meta‐analysis to detect or reject a certain intervention effect) and cumulative *Z*‐curve's breach of trial sequential monitoring boundaries.^60^

For dichotomous outcomes, we will estimate the needed data size based on observed proportion of patients with an outcome in the control group, a relative risk reduction of 25%, an alpha of 5%, and a beta of 10%. For continuous outcomes, we will in the Trial Sequential Analysis use the observed SD, a mean difference of the observed SD/2, an alpha of 5%, and a beta of 10%.

## 7 SUBGROUP ANALYSIS

Subgroup analyses will be performed per:

1. specific drugs administered;
2. trials at high risk of bias compared to trials at low risk of bias;
3. type of acute heart failure (e.g., decompensated heart failure, pulmonary edema, and de novo heart failure vs. pre‐existing heart failure);
4. time from admission to intervention (below vs. above 5 h);
5. blood pressure at baseline (above/below 140 mmHg);
6. LVEF at baseline (above/below 40%);
7. cause of heart failure (ischemic vs. non‐ischemic); and
8. documented pulmonary congestion or not.

We will use the formal test for subgroup interactions in Review Manager.

### 7.1 “Summary of Findings” table

We will create a “Summary of Findings” table using each of the primary and secondary outcomes. First, we will present our results in the “Summary of Findings” table based on the results from the trials with low risk of bias. Second, we will present the results based on all trials. We will use the five GRADE considerations (consistency of effect, bias risk of the trials, imprecision [assessed by Trial Sequential Analysis], indirectness, and publication bias) to assess the certainty of evidence.

## 8 DISCUSSION

Acute heart failure affects millions of people each year and vasodilators have been a central part of treatment for over 25 years. However, 2021 European guidelines have downgraded the use of vasodilators due to recent studies of vasodilators failing to show a benefit of this drug class. Several reviews have been made in this area, but no previous systematic reviews adhered to the PRISMA guidelines, used Trial Sequential Analysis, or have included trials from the last 5 years. Furthermore, no recent reviews are planned or published.

This systematic review protocol has several strengths. We have based the protocol on the PRISMA‐P checklist^61^ and based on the Cochrane Handbook for Systematic Reviews of Interventions we have pre‐defined our methodology^48^ and we account for the risk of random errors and systematic errors.

The systematic review will also have limitations. We will pool data from all trials regarding the treatment of pulmonary edema or acute decompensated heart failure using vasodilators and thus theoretically giving rise to statistical and clinical heterogeneity. However, we think that there is significant overlap between patients with acute decompensated heart failure and patients with acute pulmonary edema and the effects of vasodilation may therefore be similar in these different patient groups. If statistical heterogeneity is estimated to be high, we will in the end decide if a meta‐analysis of all trials should be avoided. We have pre‐defined several sensitivity analysis and subgroups to assess whether a given intervention effect will differ between conditions and trials. We may conduct additional subgroup analyses and sensitivity analyses to explain unexplained heterogeneity.

With this systematic review, we aim to provide clinicians with a reliable evidence synthesis adjusted for bias, sparse data, and multiple testing regarding the treatment with vasodilators for acute heart failure.

## AUTHOR CONTRIBUTIONS

**Johannes Grand**: Conceptualization, Methodology, Writing, Review & Editing, Visualization. **Olav W. Nielsen**: Methodology, Review & Editing. **Jacob Eifer Møller**: Methodology, Review & Editing. **Christian Hassager**: Methodology, Review & Editing. **Janus Christian Jakobsen**: Conceptualization, Methodology, Review & Editing.

## Supporting information


**Data S1** Supporting Information.
