# Supplementary material for: Vasodilators for acute heart failure—A protocol for a systematic review of randomized clinical trials with meta‐analysis and Trial Sequential Analysis
Source: Acta Anaesthesiol Scand. 2022 Aug 23;66(9):1156–64. doi: 10.1111/aas.14130 (PMC9542024; doi:10.1111/aas.14130)
Supplement: Supplementary file 1 — Data S1 Supporting Information. [file AAS-66-1156-s001.pdf]

**Search strategies for**
**Vasodilators for acute heart failure**
**(J Grand)**
**Preliminary search strategies prepared 14 July 2022**

**Cochrane Central Register of Controlled Trials (latest issue) in the Cochrane Library**
#1 MeSH descriptor: [Heart Failure] explode all trees
#2 MeSH descriptor: [Pulmonary Edema] explode all trees
#3 MeSH descriptor: [Dyspnea] explode all trees
#4 (((acute or decompensat* or destabili*) and (heart or cardiac or coronary) and (failure* or insufficienc*)) or ADHF or AHF)
#5 (((pulmonary or lung) and (edema* or oedema*)) or ((pulmonary or lung or systemic or heart) and congest*))
#6 (dyspnea or dyspnoea)
#7 #1 or #2 or #3 or #4 or #5 or #6
#8 MeSH descriptor: [Vasodilator Agents] explode all trees
#9 MeSH descriptor: [Antihypertensive Agents] explode all trees
#10 MeSH descriptor: [Calcium Channel Blockers] explode all trees
#11 MeSH descriptor: [Nitric Oxide Donors] explode all trees
#12 MeSH descriptor: [Nitroglycerin] explode all trees
#13 MeSH descriptor: [Isosorbide Dinitrate] explode all trees
#14 MeSH descriptor: [Relaxin] explode all trees
#15 (vasodilat* or vasoactiv* or vasorelaxant* or vasodilat* or "blood vessel dilat*")
#16 (antihypertensiv* or hypotensiv*)
#17 (calcium and (block* or antagonist or inhibitor))
#18 ("nitric oxide" and (donor* or agent* or compound* or drug*))
#19 (nitroglycerin* or (isosorbide and (mononitrat* or dinitrat*)) or sodium nitroprusside or serelaxin or relaxin or nesiritide or neprilysin or enalaprilat or hydralazine or clevidipine)
#20 #8 or #9 or #10 or #11 or #12 or #13 or #14 or #15 or #16 or #17 or #18 or #19
#21 #7 and #20

**MEDLINE Ovid (1946 to the date of the search)**
1. exp Heart Failure/
2. exp Pulmonary Edema/
3. exp Dyspnea/
4. (((acute or decompensat* or destabili*) and (heart or cardiac or coronary) and (failure* or insufficienc*)) or ADHF or AHF).mp. [mp=title, abstract, original title, name of substance word, subject heading word, floating sub-heading word, keyword heading word, organism supplementary concept word, protocol supplementary concept word, rare disease supplementary concept word, unique identifier, synonyms]
5. (((pulmonary or lung) and (edema* or oedema*)) or ((pulmonary or lung or systemic or heart) and congest*)).mp. [mp=title, abstract, original title, name of substance word, subject heading word, floating sub-heading word, keyword heading word, organism supplementary concept word, protocol supplementary concept word, rare disease supplementary concept word, unique identifier, synonyms]
6. (dyspnea or dyspnoea).mp. [mp=title, abstract, original title, name of substance word, subject heading word, floating sub-heading word, keyword heading word, organism supplementary concept word, protocol supplementary concept word, rare disease supplementary concept word, unique identifier, synonyms]
7. 1 or 2 or 3 or 4 or 5 or 6
8. exp Vasodilator Agents/
9. exp Antihypertensive Agents/
10. exp Calcium Channel Blockers/
11. exp Nitric Oxide Donors/
12. exp Nitroglycerin/
13. exp Isosorbide Dinitrate/
14. exp Relaxin/
15. (vasodilat* or vasoactiv* or vasorelaxant* or vasodilat* or "blood vessel dilat*").mp. [mp=title, abstract, original title, name of substance word, subject heading word, floating sub-heading word, keyword heading word, organism supplementary concept word, protocol supplementary concept word, rare disease supplementary concept word, unique identifier, synonyms]
16. (antihypertensiv* or hypotensiv*).mp. [mp=title, abstract, original title, name of substance word, subject heading word, floating sub-heading word, keyword heading word, organism supplementary concept word, protocol supplementary concept word, rare disease supplementary concept word, unique identifier, synonyms]

17. (calcium and (block* or antagonist or inhibitor)).mp. [mp=title, abstract, original title, name of substance word, subject heading word, floating sub-heading word, keyword heading word, organism supplementary concept word, protocol supplementary concept word, rare disease supplementary concept word, unique identifier, synonyms]
18. ("nitric oxide" and (donor* or agent* or compound* or drug*)).mp. [mp=title, abstract, original title, name of substance word, subject heading word, floating sub-heading word, keyword heading word, organism supplementary concept word, protocol supplementary concept word, rare disease supplementary concept word, unique identifier, synonyms]
19. (nitroglycerin* or (isosorbide and (mononitrat* or dinitrat*)) or sodium nitroprusside or serelaxin or relaxin or nesiritide or neprilysin or enalaprilat or hydralazine or clevidipine).mp. [mp=title, abstract, original title, name of substance word, subject heading word, floating sub-heading word, keyword heading word, organism supplementary concept word, protocol supplementary concept word, rare disease supplementary concept word, unique identifier, synonyms]
20. or/8-19
21. 7 and 20
22. (randomized controlled trial or controlled clinical trial or retracted publication or retraction of publication).pt.
23. clinical trials as topic.sh.
24. (random* or placebo*).ab. or trial.ti.
25. 22 or 23 or 24
26. exp animals/ not humans.sh.
27. 25 not 26
28. 21 and 27

**Embase Ovid (1974 to the date of the search)**
1. exp heart failure/
2. exp lung edema/
3. exp dyspnea/
4. (((acute or decompensat* or destabili*) and (heart or cardiac or coronary) and (failure* or insufficienc*)) or ADHF or AHF).mp. [mp=title, abstract, heading word, drug trade name, original title, device manufacturer, drug manufacturer, device trade name, keyword heading word, floating subheading word, candidate term word]
5. (((pulmonary or lung) and (edema* or oedema*)) or ((pulmonary or lung or systemic or heart) and congest*)).mp. [mp=title, abstract, heading word, drug trade name, original title, device manufacturer, drug manufacturer, device trade name, keyword heading word, floating subheading word, candidate term word]
6. (acute and (dyspnea or dyspnoea)).mp. [mp=title, abstract, heading word, drug trade name, original title, device manufacturer, drug manufacturer, device trade name, keyword heading word, floating subheading word, candidate term word]
7. 1 or 2 or 3 or 4 or 5 or 6
8. exp vasodilator agent/
9. exp antihypertensive agent/
10. exp calcium channel blocking agent/
11. exp nitric oxide donor/
12. exp isosorbide dinitrate/
13. exp relaxin/
14. (vasodilat* or vasoactiv* or vasorelaxant* or vasodilat* or "blood vessel dilat*").mp. [mp=title, abstract, heading word, drug trade name, original title, device manufacturer, drug manufacturer, device trade name, keyword heading word, floating subheading word, candidate term word]
15. (antihypertensiv* or hypotensiv*).mp. [mp=title, abstract, heading word, drug trade name, original title, device manufacturer, drug manufacturer, device trade name, keyword heading word, floating subheading word, candidate term word]
16. (calcium and (block* or antagonist or inhibitor)).mp. [mp=title, abstract, heading word, drug trade name, original title, device manufacturer, drug manufacturer, device trade name, keyword heading word, floating subheading word, candidate term word]
17. ("nitric oxide" and (donor* or agent* or compound* or drug*)).mp. [mp=title, abstract, heading word, drug trade name, original title, device manufacturer, drug manufacturer, device trade name, keyword heading word, floating subheading word, candidate term word]
18. (nitroglycerin* or (isosorbide and (mononitrat* or dinitrat*)) or sodium nitroprusside or serelaxin or relaxin or nesiritide or neprilysin or enalaprilat or hydralazine or clevidipine).mp. [mp=title, abstract, heading word, drug trade name, original title, device manufacturer, drug manufacturer, device trade name, keyword heading word, floating subheading word, candidate term word]
19. or/8-18
20. 7 and 19

21. Randomized controlled trial/ or Controlled clinical study/ or randomization/ or intermethod comparison/ or double blind procedure/ or human experiment/ or retracted article/
22. (random$ or placebo or parallel group$1 or crossover or cross over or assigned or allocated or volunteer or volunteers).ti,ab.
23. (compare or compared or comparison or trial).ti.
24. ((evaluated or evaluate or evaluating or assessed or assess) and (compare or compared or comparing or comparison)).ab.
25. (open adj label).ti,ab.
26. ((double or single or doubly or singly) adj (blind or blinded or blindly)).ti,ab.
27. ((assign$ or match or matched or allocation) adj5 (alternate or group$1 or intervention$1 or patient$1 or subject$1 or participant$1)).ti,ab.
28. (controlled adj7 (study or design or trial)).ti,ab.
29. (erratum or tombstone).pt. or yes.ne.
30. or/21-29
31. (random$ adj sampl$ adj7 ('cross section$' or questionnaire$ or survey$ or database$1)).ti,ab. not (comparative study/ or controlled study/ or randomi?ed controlled.ti,ab. or randomly assigned.ti,ab.)
32. Cross-sectional study/ not (randomized controlled trial/ or controlled clinical study/ or controlled study/ or randomi?ed controlled.ti,ab. or control group$1.ti,ab.)
33. (((case adj control$) and random$) not randomi?ed controlled).ti,ab.
34. (Systematic review not (trial or study)).ti.
35. (nonrandom$ not random$).ti,ab.
36. 'Random field$'.ti,ab.
37. (random cluster adj3 sampl$).ti,ab.
38. (review.ab. and review.pt.) not trial.ti.
39. 'we searched'.ab. and (review.ti. or review.pt.)
40. 'update review'.ab.
41. (databases adj4 searched).ab.
42. (rat or rats or mouse or mice or swine or porcine or murine or sheep or lambs or pigs or piglets or rabbit or rabbits or cat or cats or dog or dogs or cattle or bovine or monkey or monkeys or trout or marmoset$1).ti. and animal experiment/
43. Animal experiment/ not (human experiment/ or human/)
44. or/31-43
45. 30 not 44
46. 20 and 45

**LILACS (Bireme; 1982 to the date of the search)**
(((acute or decompensat$ or destabili$) and (heart or cardiac or coronary) and (failure$ or insufficienc$)) or ADHF or AHF) or (((pulmonary or lung) and (edema$ or oedema$)) or ((pulmonary or lung or systemic or heart) and congest$)) or (dyspnea or dyspnoea) [Words] and (vasodilat$ or vasoactiv$ or vasorelaxant$ or vasodilat$ or blood vessel dilat$) or (antihypertensiv$ or hypotensiv$) or (calcium and (block$ or antagonist or inhibitor)) or (nitric oxide and (donor$ or agent$ or compound$ or drug$)) or (nitroglycerin$ or (isosorbide and (mononitrat$ or dinitrat$)) or sodium nitroprusside or serelaxin or relaxin or nesiritide or neprilysin or enalaprilat or hydralazine or clevidipine) [Words]

**Science Citation Index Expanded (1900 to the date of the search) and Conference Proceedings Citation Index – Science (1990 to the date of the search) (Web of Science)**
#5 #3 AND #4
#4 TI=(random* or blind* or placebo* or meta-analys* or trial*) OR TS=(random* or blind* or placebo* or meta-analys*)
#3 #2 AND #1
#2 TS=((vasodilat* or vasoactiv* or vasorelaxant* or vasodilat* or "blood vessel dilat*") or (antihypertensiv* or hypotensiv*) or (calcium and (block* or antagonist or inhibitor)) or ("nitric oxide" and (donor* or agent* or compound* or drug*)) or (nitroglycerin* or (isosorbide and (mononitrat* or dinitrat*)) or sodium nitroprusside or serelaxin or relaxin or nesiritide or neprilysin or enalaprilat or hydralazine or clevidipine))
#1 TS=((((acute or decompensat* or destabili*) and (heart or cardiac or coronary) and (failure* or insufficienc*)) or ADHF or AHF) or (((pulmonary or lung) and (edema* or oedema*)) or ((pulmonary or lung or systemic or heart) and congest*)) or (dyspnea or dyspnoea))
